# Pixel-level annotations of context region for trustworthy diagnosis of Breast Lymph Node Metastasis from Histopathological WSI

**DOI:** 10.1016/j.dib.2025.112408

**Published:** 2025-12-22

**Authors:** Richa Malviya Dutta, Arif Ahmed Sekh, Prarthana Raghuram, Krishna Kiran, Shirin Dasgupta, Subhamoy Mandal, Debi Prosad Dogra, Pranab K. Dan

**Affiliations:** a Rajendra Mishra School of Engineering Entrepreneurship, Indian Institute of Technology Kharagpur, 721302 West Bengal, India; b Department of Computer Science, UiT The Arctic University of Norway, Tromso N-9037, PO Box 6050 Stakkevollan, Norway; c School of Medical Science and Technology, Indian Institute of Technology Kharagpur, 721302 West Bengal, India; d B. C. Roy Multi Speciality Medical Research Centre, Indian Institute of Technology Kharagpur, 721302 West Bengal, India; e Department of Computer Science and Engineering, School of Electrical and Computer Sciences, IIT Bhubaneshwar, 752050 Odisha, India

**Keywords:** Whole slide image, Annotations, Xml files, Multi-class, Trustworthy, Classification

## Abstract

There is a dearth of annotated images in digital pathology, and annotations are pivotal for supervised automated diagnosis. This work aims to create a set of data on breast lymph node metastatic whole slide images (WSI) that is truly valuable as it is annotated by the domain experts in a very precise and intensive manner. The annotations provided here are of rare kind that has pixel-level labels divided into three categories; first is based on the diagnostically significant tissue regions, second is background tissue and the third is artefacts present in breast lymph node metastatic WSI. High level annotations, as is provided in this work, for areas apart from the metastatic region is crucial for diagnosis as this is the context region which is critical for cancer diagnosis. Breast lymph node metastasis is a severe medical condition that requires significant efforts by pathologists to examine cancer under microscope using glass slides. Automated cancer diagnosis using WSI, the digitized versions of glass slides, needs to be trustworthy and responsible, which is ensured by involving domain experts in the process that begins with identifying diagnostically significant regions on a WSI. In this dataset, a two-stage high-value pixel-level annotation protocol is designed where selected 73 training images of Camelyon16 dataset are annotated by doctors and verified by pathologists for their accuracy. These annotations would help researchers to reliably prepare patches for further processing that can be used as training samples for extracting more such significant regions for data preparation from WSI for diagnosing metastasis. These reliable and trustworthy annotations would help to take it to the clinic from the research lab much quickly.

Specifications Table

**Table:** 

Subject	Health Sciences, Medical Sciences & Pharmacology
Specific subject area	Biomedical Engineering, histopathology, whole slide images, medical image analysis.
Type of data	xml Annotations, Binary masks of whole slide images, Python code. Raw.
Data collection	Abnormal whole slide images from Camelyon16 training dataset which comprises of H&E stained histopathological WSI of breast lymph node metastasis are annotated. The images are viewed and annotated using the Automated Slide Analysis Platform (ASAP) freely available online. Primary annotations on 73 WSI are performed by doctors, which are saved as .xml files. These annotations are cross examined and verified by an expert pathologist. The verified .xml files used for mask preparation, a few sample masks provided for reference, are used in further image analysis pipeline. The code for mask preparation is provided in the repository*.*
Data source location	The data is stored in Product Analytic and Modelling Lab at Rajendra Mishra School of Engineering Entrepreneurship, Indian Institute of Technology Kharagpur, West Bengal, India.
Data accessibility	Repository name: zenodo Data identification number: https://doi.org/10.5281/zenodo.17549031 Direct URL to data: https://github.com/MalviyaR/TAB-Annotations-WSI_SLN/tree/main/xml_files Instructions for accessing these data: None
Related research article	None

## 1 Value of the Data

• High-quality, pixel-level labels of diagnostically relevant tissues, background tissues and artefacts are the most precise type of annotations created so far for breast lymph node histopathology WSI. As per the authors’ knowledge the annotations available in databases are either for two class annotations cancer vs normal tissues or multiclass annotations where various types of cancer tissues are annotated. In this dataset precise annotations for the above mentioned three categories are provided.
• Pixel-level labels provided by experts are high-value data labels because of their accuracy and reliability. These characteristics are backbone of any supervised machine learning model for disease diagnosis.
• These precise annotations are extremely helpful in determining the context region in the diagnosis, which is otherwise neglected due to high cost of annotating these regions.
• The xml files can be used to create binary masks from the corresponding WSI to be downloaded from the dataset. The generated masks are used as template to extract patches from the corresponding regions and store them in respective folder to be used for classification enhanced by availability of context regions for classification.
• This dataset is prepared in addition to the already available metastatic region annotations in the Camelon16 database. Thus the combined annotations from Camelyon16 and our annotations would help researchers to develop more reliable and trustworthy models for breast metastasis classification.

## 2 Background

Breast cancer is the second most common cancers among woman all over the world [1]. H&E stained histopathology is the gold standard for breast cancer diagnosis [2]. Recently, digital pathology has been becoming increasingly popular for automating the diagnosis process. Cancer diagnosis is done by identifying abnormal tissue regions and on these abnormal regions the deep learning diagnostic models are predominantly trained. Models are either trained for binary classification where images are divided into normal and abnormal classes, or multiclass where image regions are divided into multiple classes based on cancer stage or type. There is dearth of annotated data in which the context region which plays a significant role in validating the presence and severity of cancers is labelled at pixel-level. In breast lymph node histopathology, for a single whole slide image, the tissue region, apart from the main lymph node which is also termed as background tissue region holds invaluable information about the cancer its history and possible proliferation [3]. Thus, the context region is extremely important for making a trustworthy and reliable diagnostic model. The classification models built on context along with the abnormal tissues bear more weightage than the models trained solely on abnormal classes.

## 3 Data Description

The original Camelyon16 is an H&E stained histopathological WSI dataset of breast lymph node metastasis. From this dataset the training set of abnormal class are downloaded, which is available on GigaScience Database [4]. Out of the total images 73 images are selected from the dataset. The original WSI from the dataset are not included because of the ethical issues. However, link to the original dataset is provided in reference section [4].

**Fig. 1:**
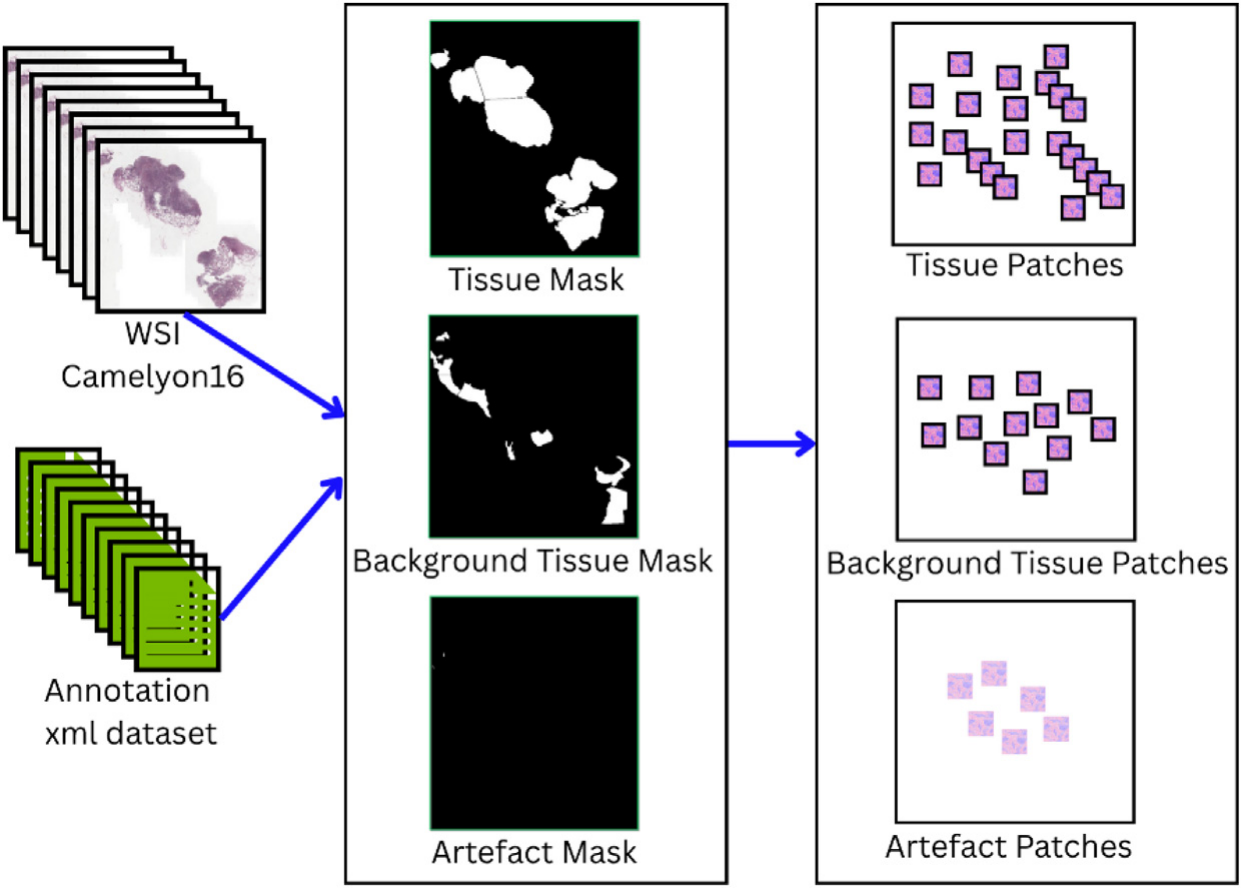
Annotations stored as xml files combined with corresponding WSI from Camelyon16 dataset used to generate masks which are further used to extract patches for training models.

The annotations for three categories, namely tissue, background tissue and artefacts are stored as separate .xml files in their respective folders, the number of xml files generated for each of the categories is mentioned in Table 1. The annotation along with the masks when combined together can be used to generate patches for three different classes in addition to annotations provided in Camelyon16 dataset as shown in Fig. 1. These patches can be used to train supervised learning model for further analysis.

**Table 1:** Number of .xml files created for the three annotated categories by domain experts.

Category	Tissue	Background Tissue	Artefact	Total
Count	73	73	63	209

Tissue region is the portion of the slide that includes the cellular mass, lymphoid tissue and tumour cells. This region also contains small amounts of adipose or other connective tissue, which is present within the lymphoid tissue mass.

**Fig. 2:**
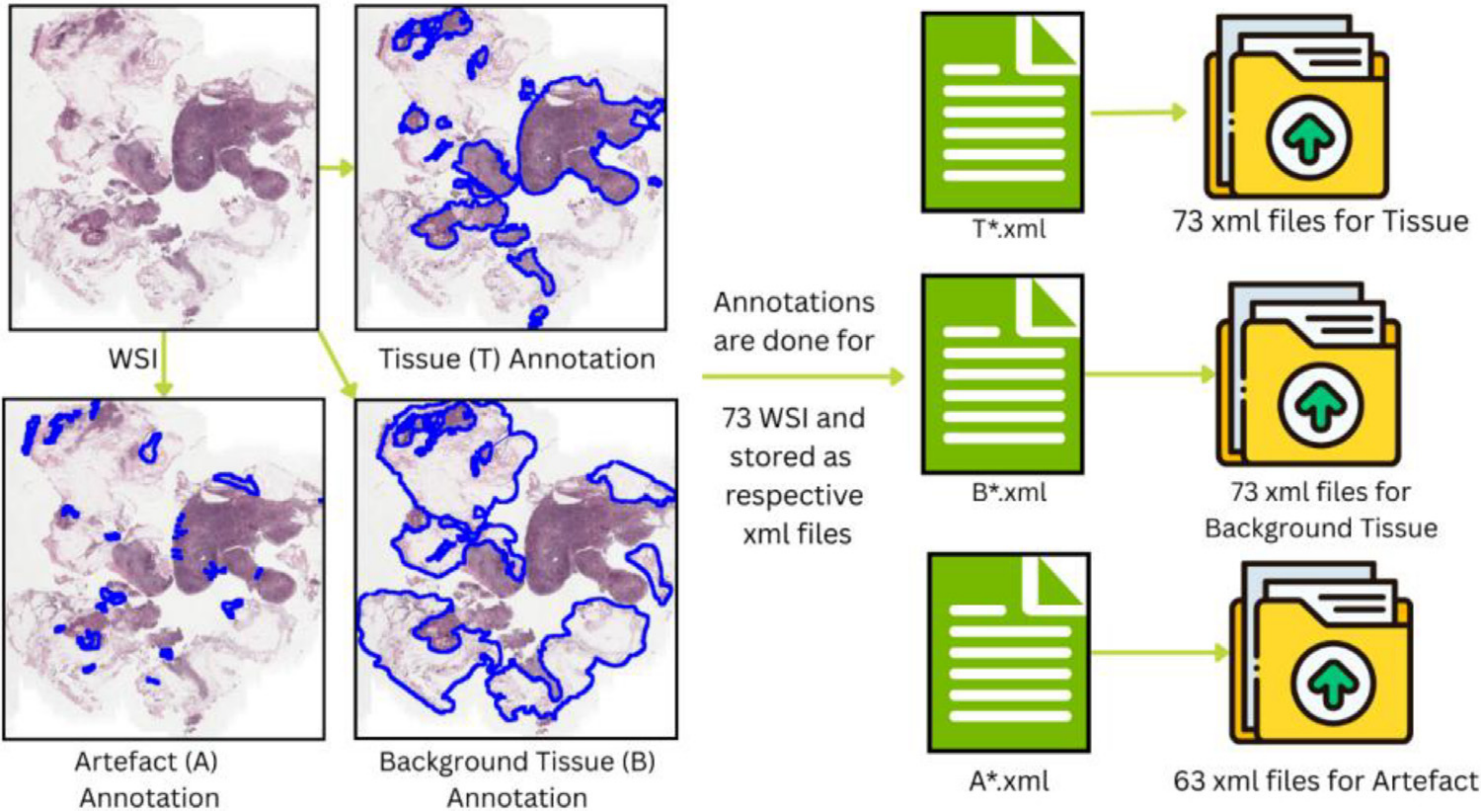
Top left corner, original WSI from Camelyon16 dataset is used to prepare annotations for Tissue (T), Background Tissue (B) and Artefact (A). These annotations are saved as their respective xml files. These files collectively are stored in three different folders belonging to their categories.

All other types of tissue visible on the slide, mainly connective tissues like blood, blood vessels, adipose tissue, etc. are marked as 'background tissue'. White background of the WSI is not annotated and is excluded. Various blemishes in slide preparation, such as dirt, hair, crushed tissue, staining artefacts which appear as dark spots or dots or lines, folds in tissue, defocused tissue either due to folds in the tissue or improper focusing while capturing WSI are annotated as “artefact”. An example of annotation is shown in Fig. 2.

A few .tiff files of the binary masks generated using .xml files are provided for reference. The python code, adapted from Camelyon16 dataset, is used for generating this binary mask is also provided in this dataset.

## 4 Experimental Design, Materials and Methods

**Fig. 3:**
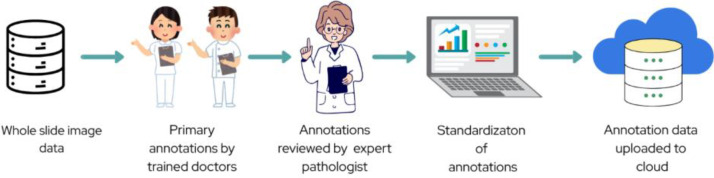
Selected WSI from Camelyon16 dataset are annotated by trained doctors which are then reviewed by expert pathologist. The annotations stored as xml files are standardized before uploading on the internet.

The annotations in the form of .xml files are obtained my providing domain experts with 73 original Camelyon16 WSI. ASAP which is a WSI viewer cum annotation tool was used, the whole experiment design is visually represented in Fig. 3.

### 4.1 Primary annotations

Medical doctors, trained to identify various tissue types, used polygon annotation tool in the ASAP software to mark regions in three categories; tissue, background tissue, and artefacts. This step is crucial to delineate precise boundaries around specific regions of the slides.

### 4.2 Reviewed annotations

A trained pathologist verified all the annotations done by doctors using same ASAP software. This verification ensures the data is reliable as pathologist have thorough understanding of objects present on the slides. Pathologist also ensures the errors present in the primary annotation stage are minimized to make the annotations more accurate and robust for downstream processing.

## Limitations

In some images the tissue region lies totally inside the background tissue region. To extract masks for only background tissue region image subtraction is needed.

This exhaustive annotation was done only on 73 WSI of abnormal class of training set. Only abnormal class is annotated because its background contains most of the context for classification.

Artefacts for the WSI is not available because of the absence or negligible presence on the slides.

The data size is small, this number maybe insufficient in dealing with various biases possibly present in the WSIs.

Annotation subjectivity could be an issue because of inter-observer variability although minimised using standard protocols and terminologies.

Large number of data points in annotation file take tremendous amount of time for mask generation.

Users need to install python based multiresolutionImageInterface library for using the code provided in the repository. Although users can implement the same using open source libraries like openslide for the same.

## Ethics statement

We utilized a public dataset and added new annotations. We confirm that the authors have read and follow the ethical requirements for publication in Data in Brief and confirming that the current work does not involve human subjects, animal experiments, or any data collected from social media platforms.

## Credit Author Statement

**Richa Malviya Dutta:** Methodology, Conceptualization, Writing-Original draft, Visualization, **Software. Sekh Arif Ahmed:** Methodology, Writing - Review and Editing. **Prarthana Raghuram:** Methodology, Data Curation, Software, Writing. **Krishna Kiran:** Methodology, Data Curation, Software, Writing. **Shirin Dasgupta:** Validation, Data Curation. **Subhamoy Mandal:** Methodology, Resources. **Debi Prosad Dogra:** Supervision, Writing – Review. **Pranab K. Dan:** Supervision, Writing – Review.

## Data Availability

zenodoTAB-Annotations-WSI_SLN (Original data)
